# Catheter malposition analysis of totally implantable venous access port in breast cancer patients

**DOI:** 10.3389/fsurg.2022.1061826

**Published:** 2023-01-06

**Authors:** Wenbo Liu, Qingzheng Han, Lin Li, Jiangrui Chi, Xinwei Liu, Yuanting Gu

**Affiliations:** The Second Department of Breast Surgery, The First Affiliated Hospital of Zhengzhou University, Zhengzhou, China

**Keywords:** totally implantable venous access port, breast cancer, ultrasound guidance, internal jugular vein, catheter malposition

## Abstract

**Background:**

To investigate the occurrence of catheter malposition in breast cancer patients undergoing Totally Implantable Venous Access Port (TIVAP) implantation and analyze the effect of TIVAP implantation site on the incidence of catheter malposition.

**Methods:**

Clinical data of Breast cancer patients underwent TIVAP implantation in our department from 2017 to 2021 was collected by reviewing the electronic medical records. The catheter malposition rate, location and management of malposed catheters in TIVAP implantation were analyzed. We divided the patients into the left internal jugular vein (IJV) group and the right IJV group according to the site of TIVAP implantation and compared the difference in the catheter malposition incidence between the two groups. In addition, we counted the catheter malposition rate of TIVAP implantion *via* the left and right IJV in right breast cancer patients to analyze the effect of tumor status on the side of TIVAP implantation on the catheter malposition rate.

**Results:**

A total of 1,510 catheters were implanted in 1,504 patients, and 16 (1.06%) had catheter malposition. The catheter malposition rate was 4.96% (7/141) for TIVAP implanted *via* the left IJV and 0.66% (9/1,369) for right IJV, with a statistically significant difference (*χ*^2^ = 18.699, *P* < 0.05). 743 TIVAPs were implanted in patients with right-sided breast tumor, of which the incidence of catheter malposition was 5.15% (7/136) for TIVAP implanted *via* left IJV and 0.82% (5/607) for right IJV, with a statistically significant difference (*χ*^2^ = 10.290, *P* < 0.05). Malposed catheters were found in the subclavian vein, IJV, brachiocephalic vein, internal thoracic vein, undefined collateral veins, and outside the blood vessels. All malposed catheters were successfully adjusted to the proper position by simple manipulative repositioning or percutaneous positioning with the assistance of digital subtraction angiography (DSA), except for 1 case was removed the port because the catheter tip was located outside the vessel.

**Conclusion:**

The catheter malposition rate of ultrasound-guided TIVAP implantation *via* IJV is low, and the malposed catheter can be successfully adjusted to the proper position by simple manipulative repositioning or DSA-assisted percutaneous positioning, however, the catheter malposition incidence of TIVAP implanted *via* left IJV is higher than that *via* the right side.

## Introduction

Chemotherapy is a necessary treatment for many cancers. Central venous access is the main way of chemotherapeutic drugs infusion. The use of Totally Implantable Venous Access Port (TIVAP) was first proposed by Niederhuber et al. (1) in 1982. Compared to the Peripherally Inserted Central Venous Catheter or Central Venous Catheter, TIVAP has become the preferred method of chemotherapy for cancer patients due to its low complication rate, long duration of use, and little effect on normal life (2–5).

The application of central venous access is often accompanied by complications such as infection, thrombosis, catheter malposition, accidental arterial puncture, pneumothorax, and cardiac tamponade (6). Catheter malposition is one of the common complications. In general, the catheter of the central venous access is often placed in the superior vena cava (SVC), and catheter malposition means that the catheter tip is not in the SVC. Some clinical practice guidelines for central venous access indicate that catheter tip position can be determined by intraoperative fluoroscopy or postoperative chest *x*-ray (7). Intraoperative fluoroscopy controls the position of the catheter tip well, but this approach requires the assistance of radiologists and the cooperation of operating room equipment, and the procedure is slightly complicated (8). It is also feasible to implant a venous port using blind insertion after ultrasound-guided access to the IJV, and taking postoperative chest *x*-ray to determine the catheter position. This technique is currently used in the most majority of Asian countries and some countries outside of Asia. However, the risk of catheter malposition in this way is higher than using intraoperative fluoroscopy. Catheter malposition may interfere with chemotherapeutic drug delivery and may lead to related complications, which should be taken seriously by clinicians. At present, few studies are focusing on catheter malposition, and the factors associated with catheter malposition are unclear. Therefore, by collecting clinical data of patients who underwent TIVAP implantation at our department, we analyzed the incidence of catheter malposition, the site and the management of malposed catheters, and tried to analyze the influencing factors of catheter malposition.

## Materials and methods

### General information

This study is a retrospective analysis of breast cancer patients undergoing ultrasound-guided TIVAP implantation *via* the IJV in the Second Breast Surgery Department of the First Affiliated Hospital of Zhengzhou University from October 2017 to July 2021. The study was conducted in accordance with the Declaration of Helsinki (as revised in 2013) and was approved by the Ethics Committee of The First Affiliated Hospital of Zhengzhou University (NO.2022-KY-0793-001).

The inclusion criteria were as follows: 1. Breast cancer patients (including patients with recurrence and metastasis); 2. Need for adjuvant chemotherapy; 3. Patients and their families consented to undergo TIVAP placement; 4. Patients were in good general condition and can tolerate TIVAP implantation. The criteria for removal from the study were as follows: 1. Auxiliary examination suggests anatomical abnormalities or thrombosis in the neck vessels; 2. Skin and soft tissue infection in the operation area; 3. Severe abnormalities in coagulation function; 4. Combination of other serious underlying diseases that cannot tolerate the procedure; 5. Patients and their families refused TIVAP implantation.

Selecting other side for venipuncture due to failed venipuncture on one side can affect the catheter malposition rate. To reduce this selection bias, we excluded cases in which the TIVAP was implanted on the other side due to a failed IJV puncture on one side. A total of 1,504 cases were included in the final analysis.

### Implantation procedures

The criteria for selecting the TIVAP implantation sites varied between the different medical groups: 1. IJV on the same side of the normal breast is preferred for surgery. 2. Clinical practice has found that the catheter malposition rate seems higher when TIVAP was implanted *via* the left IJV, so the right IJV is preferred for TIVAP implantation.

The procedures were performed by experienced surgeons in the operating room under strict sterile conditions.

Patients lay supine with heads turned to the opposite side of the operation. Ultrasound exploration of the IJV was performed to identify the puncture site. The surgical area was disinfected and covered with sterile cloth. Local infiltration anesthesia was applied to the operative area, the IJV puncture was performed under ultrasound guidance, and a guide wire was placed after the successful puncture. A subcutaneous pocket was created on the chest wall in the sub-clavicular region to place the subcutaneous reservoir. A 0.5 cm incision was made at the puncture point of the neck. A tearable sheath with a skin dilator was inserted into the IJV along the guidewire. The guidewire was removed and the intravenous catheter was placed into IJV. The length of the catheter was calculated based on the following formula: height/10–4 cm. A subcutaneous tunnel was made between the subcutaneous pocket and the neck incision using a tunneling needle, and the catheter was passed through the subcutaneous tunnel and connected to the reservoir. After confirming that the port and catheter were functional, the reservoir was secured to the chest wall with sutures. Finally, the incision was sutured with absorbable sutures.

### Imaging evaluation

The position of the catheter was observed by chest *x*-ray after the operation. As our procedures were performed without intraoperative fluoroscopy, catheter tip was acceptable as long as it was located in the SCV (that is, the catheter tip was in the range from near the right tracheobronchial angle to the right atrium), otherwise, the catheter was considered malposed. All medical imaging diagnostic reports were doubly confirmed by 2 senior radiologists.

### Data collection

Clinical data were collected by reviewing the electronic medical record, including the age, gender, tumor site, TIVAP implantation site, postoperative imaging information (whether catheter malposition occurred, the location of the malposed catheter), and the management of the malposed catheters.

### Statistical analysis

SPSS21.0 was used for statistical analysis. Age was expressed by mean ± standard deviation (Mean ± SD). Continuous correction *χ*^2^ tests were used for statistical analysis to compare the difference in the catheter malposition rate between TIVAP implantation *via* the left and right IJV, and the effect of tumor status on the side of TIVAP implantation on the incidence of catheter malposition. The difference was statistically significant when the bilateral *P* < 0.05.

## Result

### Patients characteristics

1,504 patients were enrolled in this study, of which 6 patients underwent TIVAP implantation twice, and a total of 1,510 catheters were implanted. The specific clinical characteristics were shown in Table 1, and the detailed data of 1,510 TIVAP implantation were shown in Table 2.

**Table 1:** Patients’ characteristics (*N* = 1,504).

Characteristics	*N* (%)
Age (years) (mean ± SD)	49.1 ± 9.5
**Gender**	
Female	1,497 (99.5)
Male	7 (0.5)
**Tumor site**	
Right	740 (49.2)
Left	740 (49.2)
Bilateral	24 (1.6)

**Table 2:** Details of TIVAP implantation (*N* = 1510).

Characteristic	*N* (%)	Implanted site
		Right IJV, *N* (%)	Left IJV, *N* (%)
TIVAP implanted on patients with right breast cancer	743 (49.2)	607 (81.7)	136 (18.3)
TIVAP implanted on patients with left breast cancer	743 (49.2)	738 (99.3)	5 (0.7)
TIVAP implanted on patients with bilateral breast cancer	24 (1.6)	24 (100)	0 (0)
Number	1,510 (100)	1,369 (90.7)	141 (9.3)

### Analysis of catheter malposition results

The postoperative chest *x*-ray results showed that 16 of the 1,510 cases had catheter malposition, the catheter malposition rate was 1.06%, of which the catheter malposition rate of TIVAP implantation *via* the left and right IJV was 4.96% (7/141) and 0.66% (9/1,369), respectively. According to statistical calculation, the difference was statistically significant (*χ*^2^ = 18.699; *P* < 0.05); The catheter malposition rate in 743 patients with right breast tumors was 1.62% (12/743), and the malposition rate of TIVAP implantation *via* the ipsilateral and contralateral IJV of the tumor was 0.82% (5/607) and 5.15% (7/136) respectively, the difference was statistically significant (*χ*^2^ = 10.290; *P* < 0.05). Detailed data are shown in Table 3.

**Table 3:** Catheter malposition rate.

Details	*N* (%)
Total of catheter malposition	16 (1.06)
Catheter malposition of right IJV catheterization	9 (0.66)
Catheter malposition of left IJV catheterization	7 (4.96)
malposition of right tumor catheterization	12 (1.62)
Catheter malposition of right IJV catheterization	5 (0.82)
Catheter malposition of left IJV catheterization	7 (5.15)

### Location and management of malposed catheter

**Figure 1:**
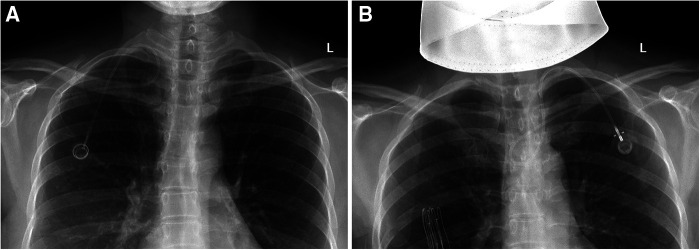
Catheter malposition to SCV. (**A**) The tip of the catheter is located in the right SCV; (**B**) The catheter turns back into the left SCV at the junction of the brachiocephalic vein and the SVC.

**Figure 2:**
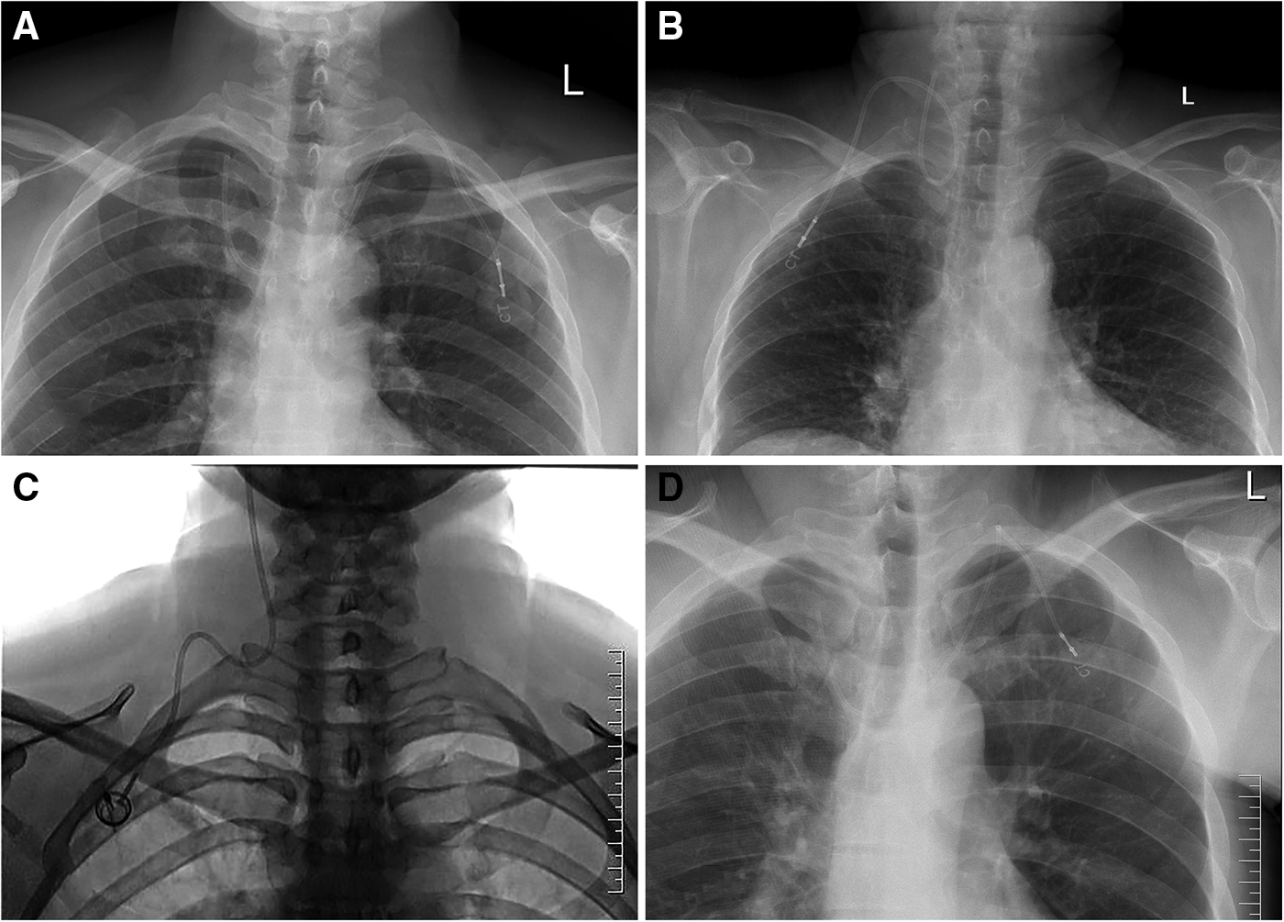
Catheter malposition to IJV and brachiocephalic vein. (**A**) The tip of the catheter is located in the contralateral IJV; (**B**) The catheter is turned back, and the tip of the catheter is located in the ipsilateral IJV; (**C**) The catheter is located in the IJV and runs toward the head; (**D**) The tip of the catheter is located in the contralateral brachiocephalic vein.

**Figure 3:**
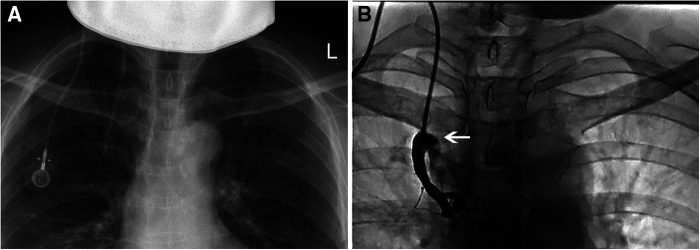
Catheter malposition to internal thoracic vein. (**A**) The chest *x*-ray suspected that the catheter was not in the SVC; (**B**) Injected the contrast medium through the port, the catheter was located in the internal thoracic vein, and part of the contrast medium flowed back into the SVC (arrow).

**Figure 4:**
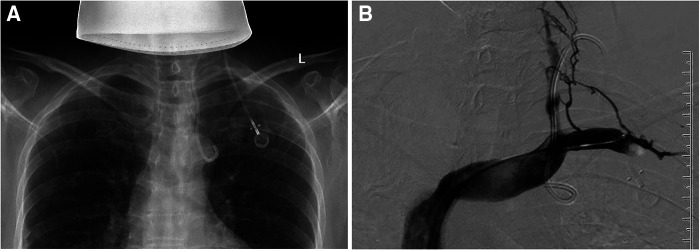
Catheter malposition to an undefined collateral vein. (**A**) Chest *x*-ray shows that the tip of the catheter is located at the aortic arch; (**B**) Fluoroscopy showed that the tip of the catheter turns back into an angle under the left clavicle, and DSA radiography shows that the vein where the catheter is located is not visible, considering that the head end enters the venule branch.

**Figure 5:**
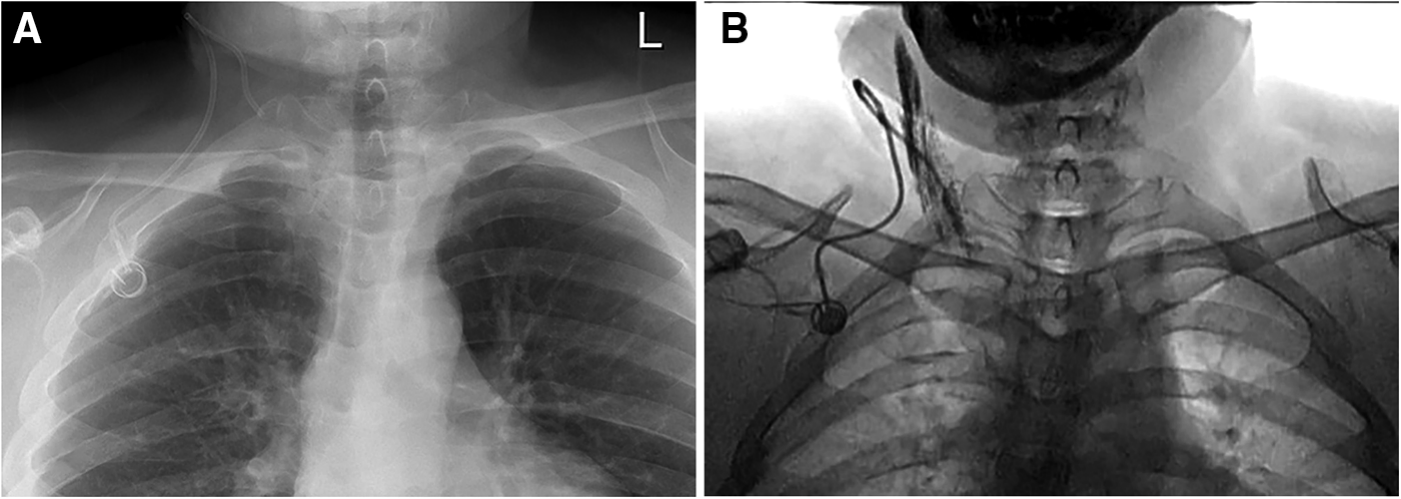
The catheter tip is located in the extravascular. (**A**) The catheter tip is seemingly located in the IJV; (**B**) No blood was drawn back from the port, and DSA shows that the contrast medium was overflowing, indicating that the catheter tip is not in the blood vessel.

The malposed catheters entered into the subclavian vein (SCV) (Figure 1), IJV (Figures 2A–C), brachiocephalic vein (Figure 2D), internal thoracic vein (Figure 3), and undefined collateral vein (Figure 4). In addition, digital subtraction angiography (DSA) showed extravascular malpositioning in 1 case (Figure 5). Except for 1 case was removed the port because the catheter tip was located in extravascular, the rest of malposed catheters were adjusted by simple manipulative repositioning or percutaneous repositioning with DSA, and all adjusted catheter tips were located in the SVC. However, the success rates differed between the two methods. The success rate of catheter adjustment by DSA was 100%, but 28.6% (2/7) of the catheters adjusted by manipulative repositioning remained malposed and were finally adjusted successfully by percutaneous repositioning with DSA. As detailed in Table 4.

**Table 4:** Details of catheter malposition and management.

Catheter malposed vessels	*N*	Treatment
Ipsilateral SCV	6	1 case was successfully adjusted by percutaneous repositioning; 4 cases were successfully adjusted by manipulation; 1 case was successfully adjusted by percutaneous repositioning after failures of the manipulation
Ipsilateral IJV	3	2 cases were successfully regulated by percutaneous repositioning and 1 case was successfully regulated by manipulation
Contralateral IJV	3	2 cases were successfully adjusted by percutaneous repositioning; 1 case was successfully adjusted by percutaneous repositioning after failing of the manipulation
Ipsilateral internal thoracic vein	1	Successfully regulated by percutaneous repositioning
Contralateral brachiocephalic vein	1	Successfully regulated by percutaneous repositioning
Undefined collateral vein	1	Successfully regulated by percutaneous repositioning
Not in the blood vessel	1	Removal of the venous access port

## Discussion

### Catheter tip position

The ideal position of the central venous catheter tip is still under debate, but it is generally considered that the lower 1/3 of the SVC or the junction of the SVC and right atrium is the desired position (7, 9, 10). Malposed catheters are often seen in the IJV, SCV, brachiocephalic vein, and right atrium; a few catheters may enter into the azygos vein, superior intercostal vein, and internal thoracic vein; the excessive length of the catheter insertion may lead the catheter into the right ventricle, coronary sinus, or even inferior vena cava (11, 12). It has been reported that malposed catheters may affect intravascular flow patterns, thereby increasing the risk of catheter-related thrombosis (2, 7); studies by Luciani et al. (13) and Schutz JC et al. (14) confirmed that higher catheter tip position is associated with a significant risk of port malfunction; complications such as arrhythmias, cardiac tamponade and cardiac perforation may occur when the catheter tip is located in the right atrium or deeper (6, 8, 11, 12, 14–16). Although guidelines issued by the National Kidney Foundation indicate that the catheter tip located in the right atrium can ensure optimal blood flow, accelerate the diffusion of chemotherapeutic drugs through the blood, and further reduce the risk of vascular injury (17), this position is often used in dialysis treatment, for the infusion of chemotherapeutic drugs, the catheter can meet the treatment demand when placed at the distal of the SVC.

### Catheter malposition rate

The catheter malposition rate of TIVAP implantation is about 0.3%–10% (3, 4, 16, 18, 19). The rate of catheter malposition in our study is 1.06%, which is consistent with previous studies. The catheter malposition rate varied with the methods of TIVAP implantation. Compared with procedures performed using body markers, the incidence of catheter malposition is lower when the procedure is performed under ultrasound guidance; if intraoperative fluoroscopy is used, catheter malposition will not occur because the catheter position can be adjusted timely; in addition, studies have shown that the catheter malposition rate of TIVAP implantation *via* SCV is usually higher than that *via* IJV, which may be related to the anatomical relationship between SCV, IJV and SVC (20).

Only a few scholars have compared the catheter malposition incidence of TIVAP implantation *via* the left and right IJV. In a prospective study of the incidence and risk of central venous catheter malposition, PikwerA et al. (19) found that the catheter malposition incidence of TIVAP implanted through the left IJV was higher than that through the right side [3.8% (4/104) VS1.4% (14/1023)], but the difference was not statistically significant; the same result was obtained in a retrospective study by Xing Lei et al. (11) on patients undergoing chemotherapy for breast cancer. This difference in malposition rates is more likely related to the anatomical characteristics of the left and right neck vessels. Compared with the left IJV, the right IJV migrates at a relatively straighter course and shorter distance toward the ipsilateral brachiocephalic vein and SVC, and the left jugular vessel has more branches (4, 8, 21, 22), therefore, TIVAP implantation *via* the left IJV may be associated with more unsuccessful placements and catheter malposition. Our study also obtained the result that the surgery performed through the right IJV had a lower malposition rate, but our result was statistically significant. This may provide some evidence for the selection of TIVAP implantation site in clinical practice.

In some studies on TIVAP implantation in breast cancer patients, the tumor site is considered in the selection of the TIVAP implantation site, and TIVAP implantation *via* the IJV contralateral to the tumor is preferred (2, 18). Some medical groups in our department also make this selection. However, few studies have analyzed the effect of the tumor status of the TIVAP implanted side on the incidence of catheter malposition in breast cancer patients. In this study, we analyzed the rate of catheter malposition in patients with right breast cancer implanted with TIVAP at different sites in our center (Left breast cancer patients were not included in this study because the right IJV is the preferred puncture vein for them, whether in consideration of tumor site or the clinical experience of the lower catheter malposition rate in right operation. This results in few left breast cancer patients undergoing surgery through the left IJV, preventing statistical analysis.). The result suggested that for patients with right breast cancer, the catheter malposition rate for TIVAP implantation *via* the left IJV was similarly higher than that for the right side[5.15% (7/136) vs. 0.82% (5/607)], and the difference was statistically significant, so we believed that the tumor status on the TIVAP implanted side had little effect on the incidence of catheter malposition. Therefore, in TIVAP implantation in breast cancer patients, the tumor site can be ignored and the right IJV is the first choice for the operation to reduce the incidence of catheter malposition.

### Management of malpositioned catheters

For malposed catheters, percutaneous repositioning with the assistance of DSA is the most effective way. It allows real-time observation of the catheter orientation and catheter tip position to achieve accurate repositioning. In addition, simple manipulative repositioning is also an available way and is less costly to treat. In our clinical practice, although manipulative repositioning cannot adjust the ectopic catheter to the proper position every time, it still has a high success rate, especially for simple malposition positions such as ipsilateral SCV and IJV. Therefore, for some medical centers that cannot perform percutaneous repositioning techniques or for patients with financial difficulties, simple manipulative repositioning can be tried first. In addition, it has been shown that intracavitary electrocardiography can indirectly determine the position of the catheter tip by observing the change in P-wave height during the procedure (23), which can improve the success rate of the operation. However, this technique has not been widely used in the clinic.

This work had some limitations. First, we did not study whether other factors such as the number of IJV punctures during the procedure and other underlying diseases of the patient's comorbidities influenced the occurrence of catheter malposition. Second, the number of cases operated *via* the left and right IJV in this study varied greatly because of the different selection criteria for the TIVAP implantation site between medical groups. Third, this study only involved catheter malposition that occurred immediately after surgery, but factors such as implantation site, position change, and lifestyle habits can cause catheter movement within the vessel during the use of the port, resulting in catheter malposition and catheter dysfunction (24). Our study did not involve this aspect. Therefore, randomized controlled trials with large samples warrant further study.

In conclusion, the application of TIVAP implanted *via* IJV in breast cancer patients during chemotherapy is safe and effective. The catheter malposition rate is low, and the majority of malposed catheters can be successfully adjusted to the proper position using manipulative repositioning or DSA-assisted percutaneous repositioning. However, the catheter malposition incidence of TIVAP implanted *via* the left IJV is higher than that *via* the right side, therefore right IJV puncture may be preferred to reduce the incidence of catheter malposition.

## Data Availability

The raw data supporting the conclusions of this article will be made available by the authors, without undue reservation.
